# Impact of radiation dose on patient-reported acute taste alteration in a prospective observational study cohort in head and neck squamous cell cancer (HNSCC)

**DOI:** 10.1007/s11547-023-01707-5

**Published:** 2023-08-29

**Authors:** Ilaria Morelli, Isacco Desideri, Andrea Romei, Erika Scoccimarro, Saverio Caini, Viola Salvestrini, Carlotta Becherini, Lorenzo Livi, Pierluigi Bonomo

**Affiliations:** 1 Radiation Oncology Unit, Department of Experimental and Clinical Biomedical Sciences “Mario Serio”, University of Florence, Azienda Ospedaliero-Universitaria Careggi, Largo Brambilla 3, 50134 Florence, Italy; 2 Radiation Oncology Unit, Azienda Ospedaliero-Universitaria Careggi, Florence, Italy; 3 Cancer Risk Factors and Lifestyle Epidemiology Unit, Institute for Cancer Research, Prevention and Clinical Network (ISPRO), Florence, Italy; 4 Radiotherapy Department, IFCA, Florence, Italy

**Keywords:** Taste impairment, IMRT, Taste buds, Dosimetry

## Abstract

**Purpose:**

Taste alteration (TA) is a frequent acute side effect of radiation treatment in HNSCC patients. Principal aim of our study was to investigate dosimetric parameters in relation to patient-assessed taste impairment in a prospective cohort treated with intensity-modulated radiotherapy.

**Methods:**

All patients with locally advanced HNSCC and amenable to radical treatment were included. Chemotherapy-induced taste alteration scale (CITAS), EORTC QLQ-C30 and QLQ-HN43 questionnaires at baseline (T0), 3 weeks (T1) and 3 months (T2) after radiotherapy conclusion were used to assess taste impairment. Base of tongue, submandibular glands (SG), parotid glands (PG) and taste buds, along with anterior and medium third of the tongue, were considered as organs at risk and thus delineated according to consensus guidelines. The mean dose to the above-mentioned structures was correlated with patient-reported outcomes.

**Results:**

Between September 2019 and November 2020, 33 patients were recruited, 31 of which analyzed. 71% had oropharyngeal carcinoma, mostly HPV-related (60%). All were treated with tomotherapy. 77.4% had concurrent cisplatin. Mean scores of general taste alterations, global health status and dry mouth and sticky saliva were assessed. The mean doses to the anterior third, medium third and base of the tongue were 23.85, 35.50 and 47.67 Gy, respectively. Taste buds received 32.72 Gy; right and left parotid 25 and 23 Gy; right and left submandibular glands 47.8 and 39.4 Gy. At univariate analysis, dysgeusia correlated with SG mean dose (95% CI 0–0.02 *p* = 0.05) and PG mean dose (95% CI 0–0.02 *p* = 0.05); dry mouth with mean dose to anterior (95% CI 0.03–1.47 *p* = 0.04) and medium third (95% CI 0.02–0.93 *p* = 0.04) of the tongue, to taste buds (95% CI 0.06–0.96 *p* = 0.03) and to SGs (95% CI 0.06–0.63 *p* = 0.02); pain mouth with mean dose to taste buds (95% CI 0–0.02 *p* = 0.04), to SGs (95% CI 0–0.03 *p* = 0.03) and to base tongue (95% CI 0–0.02 *p* = 0.02).

**Conclusions:**

Our analysis supports the influence of dose distribution on the development of TA in HNSCC patients. The contribution of dose to taste buds and tongue subvolumes remains unclear and worthy of further investigation.

## Background

Head and neck (HN) cancer is the seventh most common malignancy worldwide, accounting for 890,000 new cases and 450,000 deaths [1]. New advances in the surgical field and in RT technologies allowed improvement in survival outcomes in HN cancer patients. In particular, the spread of intensity-modulated radiotherapy (IMRT) and the concomitant use of chemotherapy showed promising results in the natural development of the disease [2]. IMRT technique already proved its crucial role in giving priority to OARs such parotid glands and constrictor muscles, thus reducing the incidence of acute and late xerostomia and dysphagia [3–6]. Among HN cancer patients, dysgeusia is a common radiation-induced side effect, with almost 75% of patients undergoing radiation treatment reporting taste dysfunction [7]. Dysgeusia may affect nutritional status and overall quality of life with a compromised ability of enjoying food and social interactions. It is associated with greater weight loss [8] and affects almost all patients during or at the end of treatment [9] with persistence in some cases [2, 10]. Not enough literature data are available to define the possible association between dose to the OARs and taste impairment, also because gustatory OARs are yet to be precisely defined. A reduction in dose to the oral cavity, with a particular focus on the anterior two-thirds of the tongue, may be associated with improvement in toxicity outcomes. Since there is not a consensus on dose constraints for the reduction of dysgeusia [11, 12], the primary aim of our monocentric experience was to investigate the impact of radiation dose to the taste bud bearing tongue mucosa, first considered as an OAR, on acute taste alterations (TA).

## Materials and methods

### Patients and treatment

This was a prospective observational cohort study conducted at Radiation Oncology Unit, University of Florence, Italy.

**Fig. 1:**
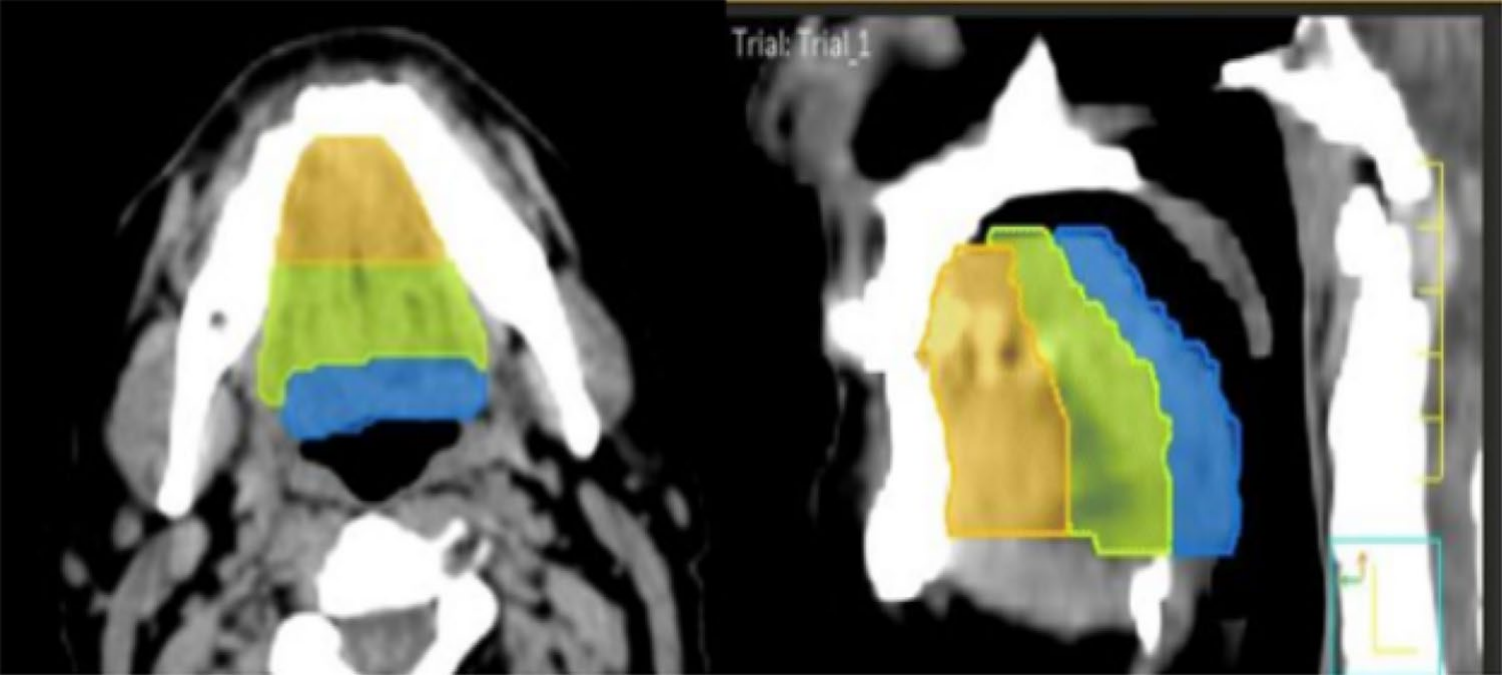
Contouring of the base (blue), medium third (green) and anterior third (yellow) of the tongue in axial (left) and sagittal (right) view on planning CT scan

All included patients had a histological diagnosis of invasive HNSCC (staged according to American Joint Committee on Cancer 8th edition) amenable to radical treatment with photon IMRT. Patients were excluded if they had locoregional or distant recurrence, other primary tumors or if they had received previous systemic treatment for metastatic disease. Also, the impossibility of undergoing planning CT, a history of major oral surgery or oral tongue devices for immobilization during radiotherapy were exclusion criteria. During planning CT and treatment, a 5-point thermoplastic mask was used to immobilize the patients. The treatment consisted of 6 MV photons with IMRT technique. Three types of schedules were allowed: 70.0 Gy in 33 fractions, 70.0 Gy in 35 fractions and 66 Gy in 33 fractions. Only patients with well-lateralized tonsil cancer could undergo unilateral RT. Otherwise, bilateral neck RT was applied. Three different parts of the tongue were considered in the contouring phase for each patient: anterior, medium third and base of tongue (Fig. 1). The taste buds volume was contoured as an OAR on the planning CT following the latest edited guidelines [11] according to which it results from an axial elaboration of a midsagittal contour. Based on Brouwer et al. work [12], ipsi- and contralateral major salivary glands were contoured. Data about irradiated volume, mean dose (Dmean), minimum (Dmin) and maximum dose (Dmax) of the above-mentioned structures were reported.

Concurrent systemic therapy was mostly platinum-based (weekly 40 mg/mq or three-weekly 100 mg/mq); targeted therapy was mainly represented by cetuximab. Induction chemotherapy was permitted, as well as adjuvant chemotherapy after concomitant RTCT (as per clinical practice and in those patients already enrolled in the DUCRO phase I/II study [13]) and radiotherapy alone without concurrent chemotherapy.

### Toxicity evaluation

Patient-reported outcomes in terms of taste alterations were prospectively assessed during and after treatment. All included patients filled in EORTC Core Quality of Life questionnaire (QLQ-C30) and EORTC Quality of Life Questionnaire Head and Neck Cancer (EORTC QLQ-H&N43). They were also asked to fill in CiTAS (Chemotherapy-Induced Taste Alterations) self-administered questionnaire which consists of 18 items exploring qualitative (heterogeusia, cacogeusia) and quantitative (hypogeusia and ageusia) alterations and problems related to nutritional intake (difficulty in eating hot and fat food). CiTAS also evaluates the four dimensions of gustatory alterations using a Likert scale 1–5 (where 1 means absence of symptoms and 5 means maximum difficulty or disturbance): intensity of taste, discomfort, phantogeusia and parageusia and general alterations of taste. This allows to have a better perception of patients’ QoL and to cope with the side effects of oncological therapies.

### Statistics

Primary endpoint was the correlation between the development of acute moderate-to-severe taste alterations and dosimetry to taste bud mucosa, considered as an OAR. SPSS Statistics version 24 (IBM statistics, Armonk, NY, USA) was used for analysis.

We performed a descriptive analysis including mean values, range (min–max) and interquartile range (IQR), parameters used to characterize overall population, taste alterations, dry mouth and pain mouth and the dose received by the OARs (major salivary glands and taste bud bearing tongue mucosa, mainly).

To verify correlation between taste impairment (from moderate to severe grade) and different variables we performed a univariable logistic Cox regression analysis. To identify correlation between the CiTAS scale and all four symptom classes (no/mild/moderate/severe) an ordinal logistic regression analysis was performed. We estimated a significant *p* value of 0.05 for this hypothesis-generating study.

## Results

### Study cohort (Table 1)

**Table 1:** Patients characteristics

Characteristics	*N* (%)
*Total patients*	31
Males	19 (61)
Females	12 (39)
*Age (years)*	
Mean	63.35
Range	49–86
*Tobacco exposure*	
Never smoker	4 (13)
< 10 p/y	4 (13)
10–20 p/y	8 (26)
> 20 p/y	15 (48)
*Tumor primary site and subsite*	
Larynx	4 (13)
Sovraglottic	1 (25)
Glottic	3 (75)
Nasopharynx	3 (10)
Oropharynx (O)	22 (71)
Base tongue	8 (36)
Soft palate	2 (9)
Tonsil	12 (55)
Hypopharynx (H)	2 (6)
Pyriform sinus	2 (100)
*Histology*	
Squamous cell carcinoma (SCC)	30 (97)
Non-keratinizing indifferentiated	1 (3)
*HPV status*	
Positive	13 (42)
Negative	10 (32)
Not assessed	8 (26)
*T stage*	
T1	8 (26)
T2	10 (32)
T3	5 (16)
T4	3 (10)
T4a	5 (16)
T4b	0 (0)
*N stage*	
N0	8 (26)
N1	12 (39)
N2	1 (3)
N2a	2 (6)
N2b	1 (3)
N2c	2 (6)
N3	2 (6)
N3a	1 (3)
N3b	2 (6)
*Stage group*	
I	5 (16)
II	6 (19)
III	8 (26)
IVa	9 (29)
IVb	3 (10)

Between September 2019 and November 2020, 33 patients were included. All patients provided written informed consent. Two patients were excluded from the present analysis because one did not receive the pre-planned intervention and the other one was lost to follow-up and never completed questionnaires at T2 time-point. Thirty-one patients were evaluated for the present analysis, 12 female (39%) and 19 male (61%). Mean age at diagnosis was 63 years (range 49–86). Four patients (13%) were never-smoker, whereas 15 patients (48%) smoked more than 20 pack/year. Most patients (*n* = 22, 71%) had oropharyngeal carcinoma, four patients (13%) had laryngeal carcinoma, three (10%) had nasopharyngeal carcinoma and two patients (6%) had hypopharyngeal carcinoma. Among oropharyngeal cancer patients, 13 (60%) were HPV-positive. Seven patients were categorized as T1 (23%), T2 in 11 (33%), T3 in 5 cases (16%) and T4 in 8 (26%). Twenty-two patients (71%) had pathologic neck nodes.

### Treatment (Table 2)

**Table 2:** Treatment characteristics

*Type of treatment*	
RTCT + CT	6 (19)
RTCT	18 (58)
RT alone	7 (23)
*Concomitant chemotherapy*	
Weekly cisplatin 40 mg/mq	1 (4)
Tri-weekly cisplatin 100 mg/mq	17 (71)
Cetuximab	1 (4)
DUCRO (cetuximab/durvalumab) [13]	5 (21)
*RT dose (Gy)*	
69.96	30 (97)
66	1 (3)
*Neck irradiation*	
Unilateral	6 (19%)
Bilateral	25 (81%)
*RT technique*	
Tomotherapy	31 (100)
*PTV (cc)*	
Mean	105
Range	23–298

Six patients (19%) received radio-chemotherapy association followed by chemotherapy (nasopharyngeal cancer patients as per clinical practice and oropharyngeal cancer patients according to phase I/II DUCRO study [13]); the majority (18 patients, 58%) underwent radiotherapy and concomitant chemotherapy and 7 cases (23%) were treated by means of radiotherapy only. No one underwent ICT before treatment. Between those who underwent concomitant chemotherapy, the most administered drug was three-weekly cisplatin 100 mg/mq (17 patients, 94%). One patient underwent cetuximab and another one received weekly cisplatin 40 mg/mq. Five patients were also enrolled in phase I/II DUCRO [13] and according to protocol study they received cetuximab and anti-PDL1 durvalumab concomitantly with radiotherapy. All patients were treated with tomotherapy with a three-dose level Simultaneous Integrated Boost (SIB) technique (69.9, 59.4 and 52.8 Gy in 33 fractions). Most patients (25, 81%) underwent bilateral neck irradiation, whereas 6 cases (19%) where treated with unilateral neck irradiation. Mean prescribed dose to the high dose planning target volume (PTV) was 69.45 Gy and mean PTV was 105 cc. Mean dose received by the extended oral cavity was 36 Gy. Mean dose to base-tongue, medium third and anterior third was 47.67, 35.50 and 23.85 Gy, respectively. The taste buds received a median dose of 32.72 Gy. For what concerns major salivary glands, right and left parotid were treated for a median fractionated total dose (FTD) of 25 and 23 Gy; right and left submandibular glands 47.8 and 39.4 Gy.

### Taste impairment assessment

All patients were asked to fill in CiTAS (Chemotherapy-induced Taste Alteration Scale), EORTC QLQ-H&N43 and EORTC QLQ-C30 questionnaires at baseline, 20 days after radiotherapy conclusion (T1) and at 3 months later (T2). CiTAS subdomains (general taste alterations, decline in basic taste, discomfort and phantogeusia/parageusia) at baseline reported a score of 1.17, 1.30, 1.28 and 1.14, respectively. At T1 they were 2.59, 3.01, 2.01 and 2.65, whereas at T2 the scores were reported to be 1.83, 2.19, 1.53 and 1.78 (Table 3). Global health status was evaluated by means of items 29 and 30 of QLQ-C30. At baseline it was scored 69.35, at T1 62.37 and at T2 71.39. QLQ-H&N43 evaluated several items; for our analysis, we considered mouth dryness and saliva thickness. At baseline, T1 and T2 it was scored 11.29, 49.46 and 46.67, respectively.

**Table 3:** Mean scores of CiTAS subdomains, dry mouth and sticky saliva and QoL at T0, T1 and T2

	T0	T1	T2
Taste alteration (CiTAS)	1.17	2.59	1.83
Phantogeusia and parageusia (CiTAS)	1.14	2.65	1.78
Taste discomfort (CiTAS)	1.28	2.01	1.53
Taste reduction (CiTAS)	1.3	3.01	2.19
Dry mouth and sticky saliva (H&N43)	11.29	49.46	46.67
Global health status (C30)	69.35	62.37	71.39

### Correlation of taste impairment and dose

**Fig. 2:**
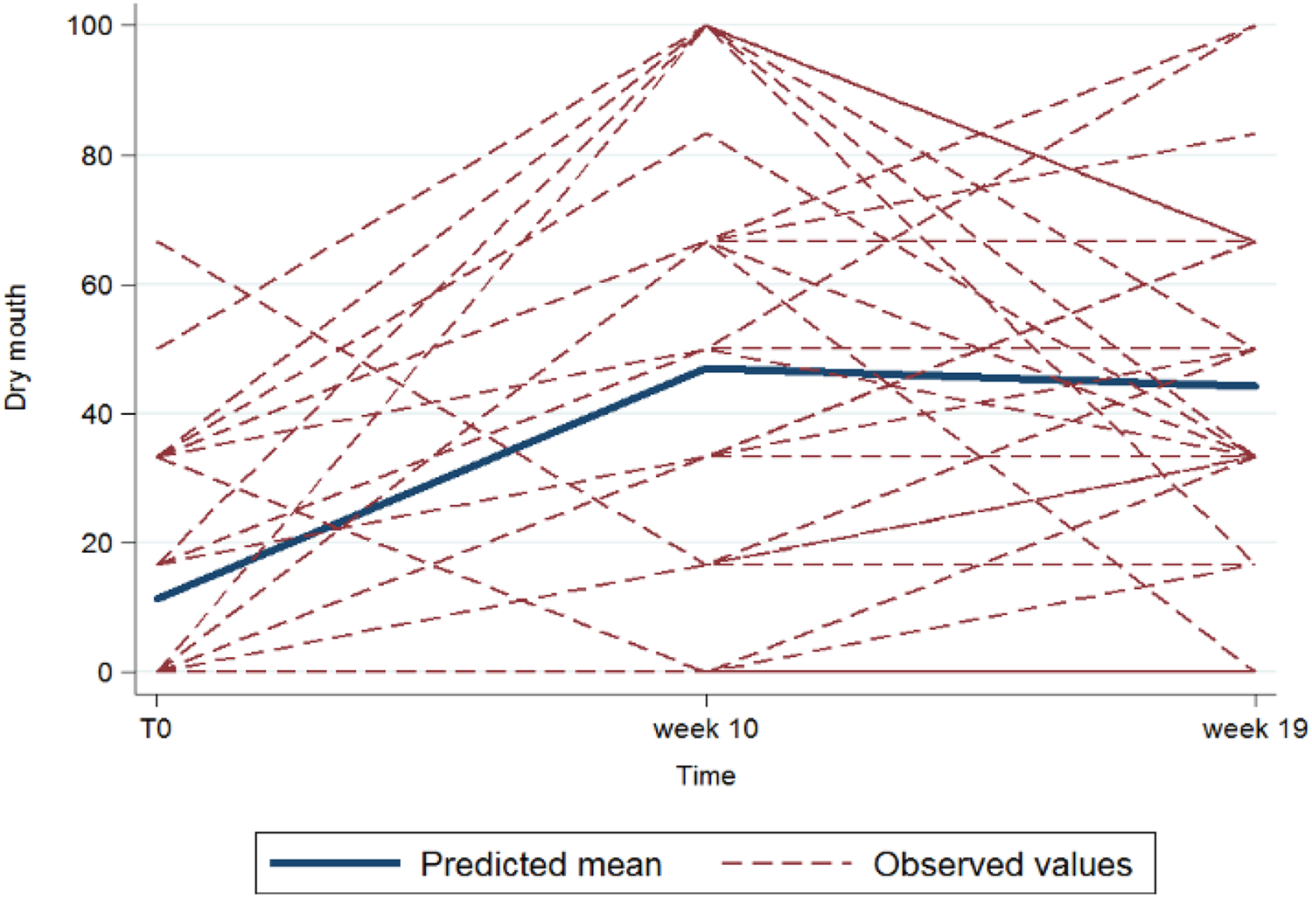
Trend over time (in weeks, abscissa axis) of dry mouth (on a scale from 0 to 100, ordinate axis). The straight blue line represents predicted mean values at baseline, w + 10 and w + 19; the dotted red lines represent the observed values in the analysis population at the same time interval and how they differ from the predicted model

**Fig. 3:**
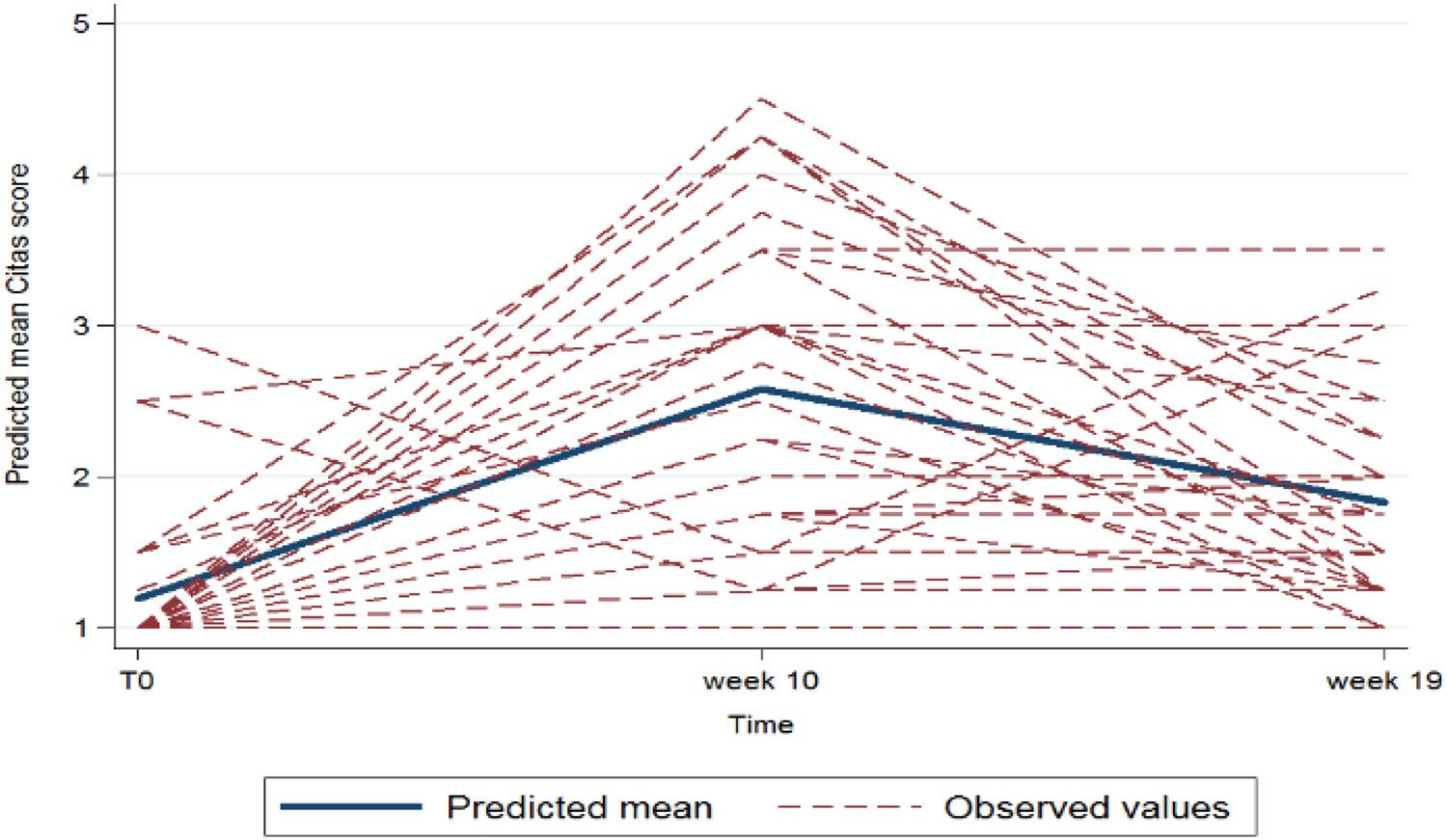
Trend over time (in weeks, abscissa axis) of CiTAS score (on a scale from 1 to 5, ordinate axis). The straight blue line represents predicted mean values at baseline, w + 10 and w + 19; the dotted red lines represent the observed values in the analysis population at the same time interval and how they differ from the predicted model

At univariate analysis (Table 4), we found a correlation between taste alteration and submandibular glands mean dose (95% CI 0–0.02 *p* = 0.05) and parotid glands mean dose (95% CI 0–0.02 *p* = 0.05); dry mouth was related to mean dose to anterior (95% CI 0.03–1.47 *p* = 0.04) and medium third (95% CI 0.02–0.93 *p* = 0.04) of the tongue, to taste buds (95% CI 0.06–0.96 *p* = 0.03) and to submandibular glands (95% CI 0.06–0.63 *p* = 0.02); pain mouth correlated with mean dose to taste buds (95% CI 0–0.02 *p* = 0.04), to submandibular glands (95% CI 0–0.03 *p* = 0.03) and to base tongue (95% CI 0–0.02 *p* = 0.02). The trend over time of dry mouth and CITAS score is reported in Figs. 2 and 3.

**Table 4:** Univariate analysis for taste alterations, dry mouth and pain mouth

Univariate (i.e., one variable at a time)	CITAS (taste alterations)				HN43-dry mouth (linearly transformed)				HN43-pain mouth			
Results for + 100 cGy increase in medium dose	Coeff.	Lower 95%CI	Upper 95%CI	*p* value	Coeff.	Lower 95%CI	Upper 95%CI	*p* value	Coeff.	Lower 95%CI	Upper 95%CI	*p* value
Base tongue	0.01	− 0.01	0.02	0.248	0.33	− 0.07	0.72	0.107	0.01	0	0.02	0.023
Medium third	0.01	0	0.02	0.109	0.48	0.02	0.93	0.04	0.01	0	0.02	0.101
Anterior third	0.01	− 0.01	0.03	0.326	0.75	0.03	1.47	0.042	0.01	− 0.01	0.03	0.314
Taste buds	0	− 0.01	0.02	0.71	0.51	0.06	0.96	0.028	0.01	0	0.02	0.038
Parotid glands	0.03	0	0.05	0.051	0.62	− 0.33	1.57	0.202	0	− 0.02	0.03	0.808
Submandibular glands	0.02	0	0.04	0.053	0.66	0.12	1.19	0.017	0.02	0	0.03	0.029

## Discussion

The principal purpose of our analysis was to investigate the influence of dosimetric parameters on the development of acute dysgeusia in HN cancer patients treated with IMRT. The taste bud bearing tongue mucosa was firstly identified as an OAR and we also provided an anatomically delineated division of the tongue into three substructures, instead of considering the entire tongue or the whole oral cavity, for our dosimetric analysis of radiation-induced taste alterations.

We could not find a correlation between dose to taste buds and dysgeusia, but at univariate analysis dose to taste buds was positively associated with dry mouth and pain mouth. We found instead a significant positive relation between acute TA and mean dose to submandibular and parotid glands, thus showing that dose to salivary glands might play a more crucial role in the development of radiation-induced TA, compared to the dose to the taste buds. In line with our findings, in their recent work, Stieb et al. outlined the correlation between dosimetric parameters of major salivary glands and the progression of late taste impairment with no significant influence of the dose to taste buds on dysgeusia [14].

Most patients experience partial or total taste loss in all five tastes during radiotherapy (RT), with maximum impairment between the fourth and the sixth week [15] and with taste recovery 4–12 months after the end of RT [16]. In this regard, in 2019 Martini et al. prospectively evaluated the development of dysgeusia in a cohort of 31 HN cancer patients treated with either exclusive radiotherapy or combined modality treatment. According to their results, a progressive increase in all dysgeusia dimensions was detected, with a peak at the seventh week of treatment. A partial recovery was highlighted after the end of RT, with heterogeneities according to different dysgeusia dimensions. Indeed, a recovery in terms of discomfort, phantogeusia/parageusia and general taste alterations was observed at the sixth month observational time point, while a significant difference could still be observed at a later stage in terms of hypo-ageusia [17]. Although we could not prove a correlation between median dose to the taste buds and dysgeusia, taste impairment during RT was historically considered the effect of taste buds’ atrophy. Mossman first studied purified taste cell membranes from bovine tongues and observed that the percentage of intact taste cell membranes decreased exponentially with increasing radiation dose. Taste impairment could be then regarded as only related to direct radiation damage and the role of salivary dysfunction was not considered [18]. In Just experience, patients complaining from taste impairment during RT had many changes in epithelial cells of papilla, with thicker areas and smaller distribution of taste pores, without any change of the taste bud structure [19]. Yamashita supported the theory of taste buds’ atrophy in the determination of taste loss, as he emphasized how the main cause of radio-induced taste alterations is the loss of taste buds and not the damage to gustatory nerves [20]. Sandow, as well, showed that patients who experienced taste impairment during RT could recover by 6 months, thus indicating that radiation acts at the level of the taste cells rather than affecting the nerves [21].

A correlation between the severity of dysgeusia and dosimetric parameters to the oral cavity and to the tongue was detected by many authors. It is reported that loss of taste detection can be experienced by almost all patients at a dose of 60 Gy to the entire oral cavity [22]. Sapir et al. in their work analyzing 73 patients with locally advanced oropharyngeal cancer found that the median dose received by the oral cavity (*P* = 0.005) and the tongue (*P* = 0.019) was positively correlated with patient-reported severe dysgeusia [23]. In Chen and colleagues’ work, glossectomy (partial or total) was the only relevant factor on long-term taste impairment. With the exclusion of patients undergoing surgery, the mean doses to the parotid glands and to oral cavity were the only factors involved in the prediction of taste loss. Indeed, a mean oral cavity dose < 50 Gy was related to 10.5% of patients suffering from TI; when the mean dose was ≥ 50 Gy, TA was experienced by 38.7%, instead [16]. In Chen work of 2022 on 87 Taiwanese patients receiving HN IMRT, the dosimetric parameter most associated with taste dysfunction was a mean oral cavity dose > 40 Gy [24].

Many authors established a significant association between the extent of dysgeusia and the proportion of tongue included in the radiation field [15]. In this context, Kamprad and colleagues conducted a prospective study with the result that entire-tongue irradiation may lead to the development of major gustatory defects and longer recovery time. Therefore, reduction of the highly exposed tongue volume by IMRT is recommended [25]. In another study by Yamashita, which included 118 patients, radiation to the anterior part of the tongue was the most important predictor of acute taste loss, regardless of the inclusion of base tongue in the high-dose region. Even low radiation doses (such as 20 Gy) can determine temporary taste loss if the anterior part of the tongue is irradiated [26]. Therefore, taste impairment, malnutrition and gastrotomy tube dependency could be reduced by preserving from radiation damage as much as possible of the anterior third of the tongue/oral cavity, thus improving patients’ overall quality of life and survival [15].

We did not evaluate the impact of concurrent chemotherapy on dysgeusia and this can be considered a limitation of our analysis; on the contrary, Stieb and colleagues show that in patients undergoing chemotherapy severe TA was detected less often, although without a statistically significant difference [14]. Other limitations of our study must be emphasized. We performed our analysis on a small sample size and we did not stratify our population according to smoke habit, alcohol consumption and HPV status, which may have affected taste impairment at baseline. We only considered acute taste impairment with questionnaires completion at baseline, three and six months after the end of RT. Other dosimetric parameters (as D95 and D5) might be more useful for taste impairment than mean doses only.

Also, in the IMRT and radiomics era, imaging may be of crucial importance in evaluating the individual predisposition to develop radiation-induced toxicity and radiomics features have been recently explored for what concerns xerostomia and dosimetry to salivary glands with the result that radiomics-based analyses can assess the risk of radiation-induced side effects in HN cancer patients [27–31]. Also, the advancement of IMRT has already showed promising results in terms of deglutition outcomes, by sparing salivary glands and other swallowing OARs [3–6, 32]. In this context dosiomics, radiomics and morphological data regarding salivary glands and the other gustatory OARs and all integrated in a machine-learning approach could be of future crucial importance in the prediction of both acute and late dysgeusia.

## Conclusions

In our analysis, dosimetry to submandibular and parotid glands significantly correlated with acute taste impairment in patients undergoing IMRT for head and neck malignancies. Mean dose to taste buds affects dry mouth and pain mouth with no significant correlation with the development of dysgeusia. As a result, the dose to salivary glands seems to affect the development of acute taste impairment more than dosimetry to the taste bud bearing tongue mucosa.
